# Local Items

**Published:** 1885-09

**Authors:** 


### LOCAL NEWS.

The Chicago Medical Society now holds its semi-monthly meetings in the Probate Court room, on Clark street, instead of in the Grand Pacific Hotel, where its meetings were held for several years.

The regular medical colleges of Chicago begin their winter course of lectures on the 22d day of September, this year.

